# Type 2 Diabetes Mellitus: A Metabolic Model of Accelerated Aging - Multi-Organ Mechanisms and Intervention Approaches

**DOI:** 10.14336/AD.2025.0233

**Published:** 2026-04-28

**Authors:** Ziran Zhang, Xiaolin He, Yuxin Sun, Jitong Li, Jia Sun

**Affiliations:** Department of Endocrinology and Metabolism, Zhujiang Hospital, The Second Clinical Medicine School, Southern Medical University, Guangzhou, 510282, China

**Keywords:** Type 2 diabetes mellitus, aging, oxidative stress, insulin resistance, advanced glycosylation end products, glucotoxicity

## Abstract

Type 2 diabetes mellitus (T2DM) is a metabolic disease characterized by chronic high blood sugar levels and insulin resistance (IR). Modern medicine has shown that diabetes plays a role in speeding up the aging process of the body independently of age, making it an age-related aging disease. The oxidative stress caused by chronic high blood sugar and IR can lead to dysfunctional mitochondria, which in turn promotes changes in epigenetic regulation, shortening of telomeres, and cellular senescence. There is currently a lot of interest in understanding how T2DM contributes to senescence. This review synthesizes epidemiological and clinical research findings on aging across various organs, focusing on insulin resistance and oxidative stress as primary mechanisms. It introduces four diabetes-specific aging axes: glucose toxicity, toxicity of advanced glycation end-products (AGEs), immunoinflammatory aging, and protein amyloidosis, which are integrated into the "metabolism-inflammation-aging" network. Additionally, we provide new insights into interventions targeting aging in diabetes.

## 1. Introduction:linking aging to diabetes

The aging population is increasingly concerned due to extended life expectancy, driven by advancements in healthcare, improved access to education, and declining fertility rates. This demographic shift necessitates innovation in healthcare. According to the World Health Organization, the global population aged 65 and above is expected to increase from 761 million in 2021 to 1.6 billion by 2050, accounting for over 16% of the total population. Furthermore, the number of individuals aged 80 years or older is expected to triple, growing from 143 million in 2019 to 426 million in 2050. Population aging is associated with a higher prevalence of chronic age-related conditions such as metabolic, musculoskeletal, cardiovascular, and neurodegenerative diseases [1]. Current research is focused on understanding the key characteristics of aging to pinpoint potential therapeutic targets that may slow down the aging process. However, it also presents opportunities for healthcare innovation to address these challenges.

Diabetes mellitus is a metabolic disorder characterized by persistent high blood sugar resulting from defects in insulin secretion, action, or both, posing a growing public health issue [2]. This is particularly true for diabetes, whose prevalence is rapidly increasing alongside the aging population. The International Diabetes Federation forecasts a 46% increase in diabetes cases worldwide by 2045 [3].

Diabetes is widely recognized as a model of accelerated aging in the human body, evidenced by the positive correlation between diabetes, telomere shortening, and epigenetic aging [4, 5]. The Melbourne Collaborative Cohort Study (MCCS) revealed that 87.5% of individuals with diabetes exhibited signs of aging over a 12-year follow-up period, 10% more than non-diabetic individuals [6]. Other aging-related effects in diabetic patients include reduced physical functioning, increased mortality, and shorter life expectancy, particularly in those with early-onset diabetes or older adults [7, 8]. Chronic hyperglycemia contributes to earlier cardiovascular events, nephropathy onset, and significantly increased mortality in diabetic individuals [9, 10]. Those diagnosed with T2DM between the ages of 20 and 40 have a reduced life expectancy by 14 years for men and 16 years for women compared to non-diabetic individuals [11]. Additionally, diabetic patients are more likely to develop age-related conditions such as visual impairment, sarcopenia, osteoporosis, cardiovascular disease, renal insufficiency and mild cognitive impairment [12]. These findings suggest that diabetes is a disease associated with accelerated aging, affecting multiple aging phenotypes, potentially driven by a common set of molecular and cellular mechanisms underlying these chronic conditions [13].

**Figure 1.**
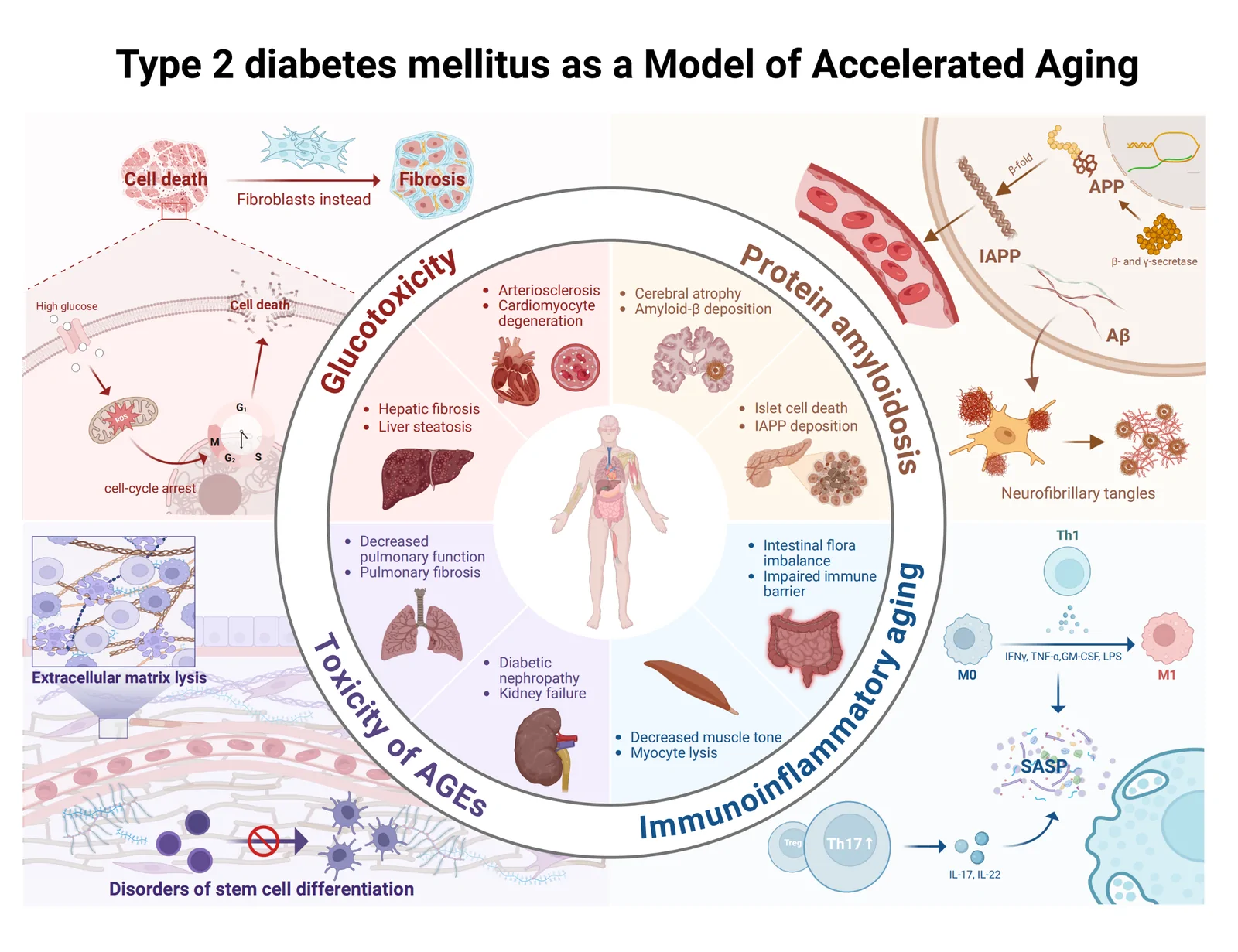
**Type 2 Diabetes Mellitus as a Model of Accelerated Aging**. This schematic outlines the organ manifestations and specific mechanisms by which T2DM promotes accelerated aging. Chronic hyperglycemia promotes advanced glycation end products, reactive oxygen species, and inflammatory cell activation (e.g., macrophage polarization to a pro-inflammatory M1 phenotype, imbalanced Th1/Th17 cell differentiation) leading to proteostatic imbalances, systemic inflammation, and immune dysregulation. ROS-mediated extracellular matrix cleavage, fibroblast activation, fibrosis, and cell-cycle arrest contribute to the generation of senescence-associated secretory phenotype. These ultimately lead to organ-specific pathologies: hepatic fibrosis, brain amyloid-β deposition, pancreatic islet cell death (via IAPP aggregation), atherosclerosis, cardiomyocyte degeneration, pulmonary fibrosis, and diabetic nephropathy. LPS: lipopolysaccharide; Aβ: amyloid-beta; APP, amyloid precursor protein; IAPP: islet amyloid polypeptide; SASP: senescence-associated secretory phenotype.

Lopez et al. summarized twelve hallmarks of aging. Among these are cellular senescence, dysregulation of nutrient sensing, epigenetic changes, telomere shortening, mitochondrial dysfunction, loss of cellular autophagy, depletion of stem cells, and chronic inflammation. These factors are strongly associated with T2DM; however, they lack specificity when it comes to diabetic aging [14]. While both aging and T2DM are linked to metabolic dysfunction, the specific mechanisms that accelerate their combined effects remain unclear in literature. Most existing reviews have focused on individual organs-such as diabetic nephropathy-or isolated pathways like oxidative stress, often overlooking a comprehensive, systems-level perspective. This review aims to synthesize findings from both epidemiological and clinical research, emphasizing trends in aging across multiple organs. It consolidates the discussion around insulin resistance and oxidative stress as key mechanisms that affect all organs. Additionally, it introduces four diabetes-specific aging axes: glucose toxicity, AGE-RAGE interactions, immunoinflammatory aging, and protein amyloidosis (Fig. 1). These axes are integrated into a unified framework known as the “metabolism-inflammation-aging” network. This framework helps explain why T2DM accelerates aging beyond traditional pathways. Furthermore, this review investigates the anti-aging effects of current diabetes therapies. By analyzing the triggers of organ-specific aging, we provide a roadmap for the targeted redesign of drugs aimed at treating the aging associated with diabetes.

## 2. Diabetes-accelerated multi-organ aging

**Figure 2.**
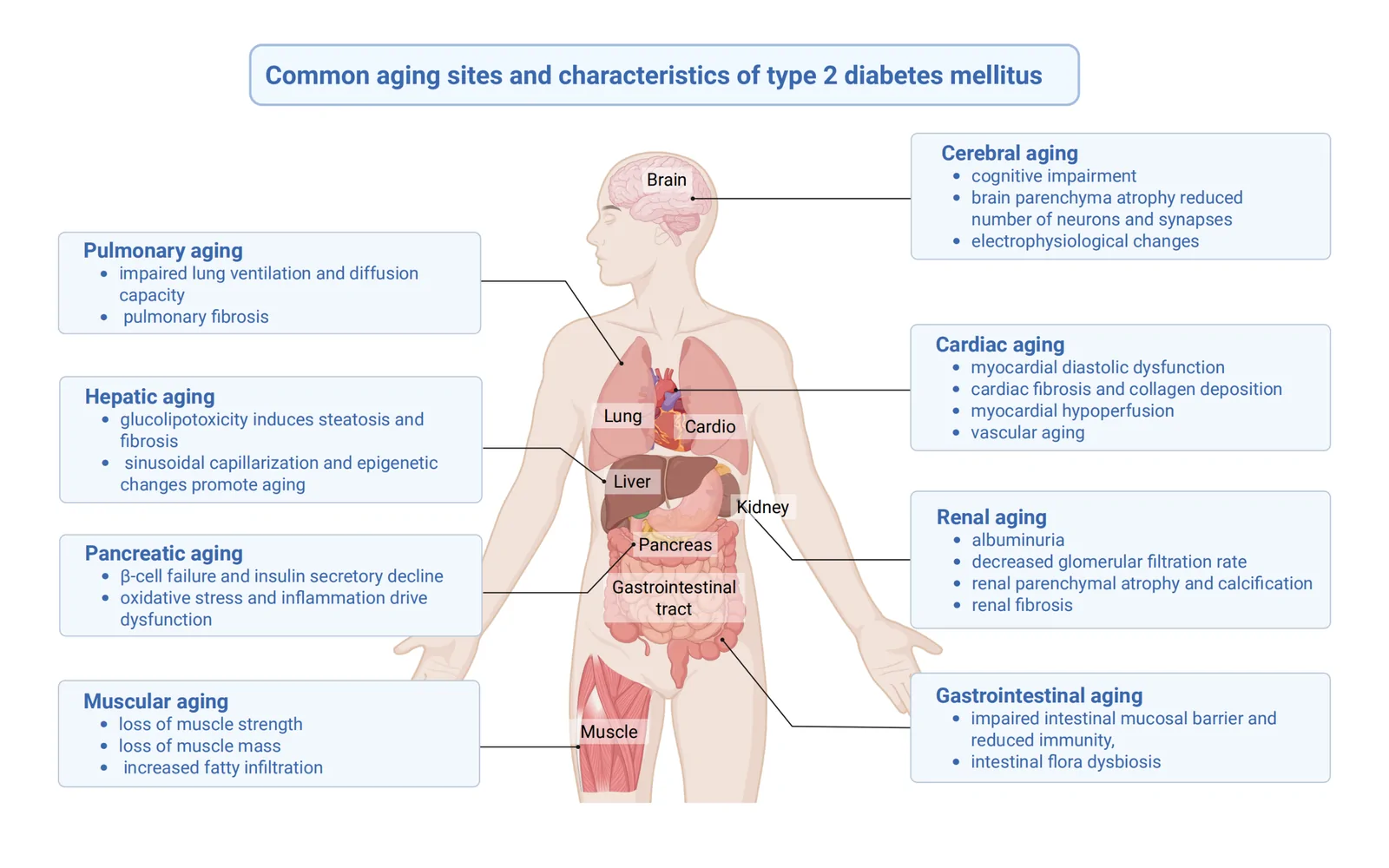
Common aging sites and aging characteristics of T2DM.

T2DM is increasingly recognized as a systemic driver of premature organ aging, characterized by accelerated functional decline across multiple organs. Key manifestations include accelerated cognitive decline, sarcopenia progression, cardiac dysfunction, pulmonary impairment, renal deterioration, gastrointestinal dysregulation, pancreatic β-cell exhaustion, and hepatic fibrogenesis. These effects stem from shared pathways (eg. IR, glucolipotoxicity, oxidative stress, and epigenetic dysregulation) that disrupt tissue homeostasis and amplify organ-specific aging patterns. Underlying molecular mechanisms involve mitochondrial impairment, dysregulated signaling, and senescence-associated secretory phenotypes (SASP). This section explores T2DM-linked accelerated aging through eight organs and four key perspectives (Fig. 2): (1) epidemiological contrasts in organ aging between diabetic and non-diabetic populations, (2) organ-specific clinical manifestations and validated senescence biomarkers, (3) quantitative evidence of accelerated degeneration rates from longitudinal cohort studies and intervention trials, and (4) mechanistic insights into T2DM-driven molecular pathways orchestrating premature aging. The analysis integrates current understanding of how diabetic pathophysiology intersects with fundamental aging mechanisms, providing a comprehensive framework for addressing diabetes-related multi-organ senescence.

### 2.1 Cerebral aging

With advancing age, the brain undergoes natural aging even in the absence of disease, manifesting as functional decline and significantly increased susceptibility to neurodegenerative disorders such as AD [13]. Notably, T2DM has been demonstrated to significantly exacerbate this physiological process. Recent neuroimaging analyses reveal reveal T2DM patients exhibit a 2.3-year brain age gap (BAG) versus chronological age, expanding to 4 years with poor glycemic control (HbA1c=8%) [15]. Pathologically, T2DM drives more pronounced gray matter loss in memory-critical regions compared to non-diabetic peers. Specifically, the ventral striatum demonstrates a 6.2% volumetric loss in diabetics, dramatically exceeding the 0.5% annual atrophy in healthy aging [16]. Longitudinal cohort studies provide dynamic evidence for this association. The China Health and Retirement Longitudinal Study (CHARLS, n=12,422) revealed 68% faster global cognitive decline and 96% accelerated visuospatial deterioration in new T2DM patients within 4 years post-diagnosis, with cognitive deterioration rates linearly correlated to diabetes duration independent of onset age [17, 18]. Complementing this, the UK Biobank study (n=31,229) quantified that T2DM patients exhibit twice the brain atrophy rate of non-diabetics, with each 1% increase in HbA1c corresponding to an additional 0.27-year BAG expansion [15]. These findings collectively establish a clear temporal association between diabetes progression and accelerated cerebral senescence.

T2DM increases cerebral aging via mechanisms different from normal senescence. Central to this is cerebral IR, where impaired PI3K/Akt signaling disrupts β-amyloid (Aβ) clearance, promoting pathogenic plaque deposition-a process that progresses decades faster than the genetically influenced slow accumulation observed in healthy aging [19]. The mechanisms of brain Aβ deposition in T2DM are described in detail in the next section. Recent genome-wide association studies (GWAS) identify shared risk loci (e.g., APOE e4, TCF7L2 rs7903146) between T2DM and neurodegeneration [20, 21]. Single nucleotide polymorphisms (SNPs) in neuroprotective genes such as SIRT1 [22] and mitochondrial quality control regulators (PINK1/Parkin) [23] demonstrate significant colocalization with diabetes-associated cognitive decline.

### 2.2 Muscular aging

Recent studies indicate that individuals with T2DM exhibit significantly accelerated muscle aging compared to non-diabetic populations. Epidemiological studies highlight a 2-3-fold higher prevalence of sarcopenia in T2DM patients versus non-diabetic individuals, with earlier onset and distinct pathological features [24]. Unlike age-related sarcopenia (type II fiber atrophy), T2DM preferentially depletes oxidative type I fibers while increasing glycolytic type II fibers, correlating with prolonged hyperglycemia and [25] IR. Younger T2DM patients also exhibit premature muscle decline, marked by reduced strength and mass. Longitudinal data reveal accelerated muscle loss in T2DM. A 16-year prospective cohort showed an annual muscle mass decline of 0.244 kg in diabetic individuals, 1.4 times faster than in non-diabetic controls [26]. Another 7-year longitudinal study, integrating metabolomics and mass spectrometry, identified distinct amino acid profiles as potential predictors of accelerated muscle aging in T2DM. These metabolic signatures correlate with impaired protein synthesis and mitochondrial dysfunction [27].

While T2DM-associated muscular aging and conventional sarcopenia exhibit overlapping pathological features including chronic low-grade inflammation and oxidative stress, IR is identified as the distinct pathogenic mechanism driving accelerated muscle deterioration in T2DM. IR hyperactivates mTORC1 (impairing autophagy and causing protein aggregation) and activates FoxO (triggering proteolysis via ubiquitin-proteasome) to accelerate muscle aging [25]. Genetic studies further identify elevated frequencies of muscle-aging-associated variants (e.g., STING) in T2DM populations. These variants may exacerbate metabolic dysfunction by targeting peroxisome proliferator activated receptors ? degradation and suppressing fatty acid oxidation, thereby limiting muscle regenerative capacity [28].

### 2.3 Cardiac aging

Emerging evidence reveals that T2DM drives multi-organ senescence. Like cerebral and muscular decline, T2DM accelerates heart aging, marked by a 4-to 8-fold higher risk of premature heart failure (HF) compared to non-diabetic populations [29]. Key aging-related cardiac pathologies, such as left ventricular diastolic dysfunction and myocardial fibrosis, are markedly exacerbated in T2DM patients, occurring decades earlier than in age-matched controls [30]. Notably, experimental evidence reveals that diabetes itself induces cardiomyocyte senescence independent of chronological age, impairing cardiac stem cell regenerative capacity [31]. A prospective study involving 4,774 pre-heart failure patients highlighted that T2DM individuals with HbA1c =7% face a 6-fold higher risk of progressing to clinical HF compared to non-diabetic counterparts [32]. Therapeutic advances demonstrate SGLT2 inhibitors reduce HF hospitalization by 30-49% [33], and trials further highlight that SGLT2 inhibitors attenuate heart aging by reversing aging-associated pathways like oxidative stress and energy dysregulation [34, 35]. These findings underscore the potential of targeted therapies to reverse pathological aging trajectories.

T2DM accelerates cardiac aging through three interlinked pathways [36]: (1) lipotoxicity / glucotoxicity-driven mitochondrial dysfunction; (2) Reactive oxygen species (ROS)-AGEs axis-mediated fibrosis; and (3) The renin-angiotensin-aldosterone system/sympathetic hyperactivity-induced calcium dyshomeostasis. Emerging genetic studies suggest polymorphisms in diabetes-associated genes (e.g., TCF7L2, PPARG) may exacerbate heart aging by impairing insulin signaling and lipid metabolism [37]. The details of the mechanism can be found in the following article. Further exploration of precise typing and early biomarkers of diabetic cardiomyopathy and optimization of comprehensive management strategies centered on SGLT2 inhibition and glucagon-like peptide-1 (GLP-1) receptor agonists are needed in the future [38].

### 2.4 Pulmonary aging

T2DM patients exhibit accelerated lung aging, marked by reduced forced vital capacity (FVC) and forced expiratory volume in 1 second (FEV1) compared to non-diabetic individuals [39]. Furthermore, elevated SASP factors (IL-6, TNF-a), shortened telomere length, and accelerated DNA methylation aging clocks in pulmonary tissues of T2DM patients collectively indicate accelerated cellular senescence in the lungs [40]. Longitudinal studies reveal T2DM adults experience 10% faster annual FVC decline compared to non-diabetic individuals (64 vs. 58 mL/year), with IR as a key predictor [41]. Metabolomic analyses in a Chinese cohort linked dysregulated lipid and amino acid metabolites in T2DM plasma to lung function decline [42]. In intervention trials, mesenchymal stem cell (MSC) therapy not only improved glycemic control but also mitigated pulmonary fibrosis and alveolar epithelial senescence through anti-inflammatory and pro-angiogenic mechanisms, though detailed pathways require further elucidation [43, 44].

Mechanistically, IR disrupts PI3K/AKT signaling, causing mitochondrial ROS accumulation and p53/p16-driven senescence in lung cells [45]. Chronic inflammation and immune dysregulation, characterized by macrophage and T-cell infiltration in diabetic lungs (akin to pancreatic islet pathology), promotes alveolar destruction and extracellular matrix deposition [46] via pro-fibrotic factors [47]. GWAS implicates T2DM-associated genes (e.g., RFX6) in impairing alveolar surfactant secretion and accelerating functional decline through ion channels dysregulation [48, 49].

### 2.5 Renal aging

Recent epidemiological studies have revealed accelerated kidney aging characteristics in patients with T2DM [50]. Compared with non-diabetic populations, T2DM patients exhibit significantly higher annual renal function decline rates (eGFR decrease of 2.5 vs 1.4 mL/min/1.73 m²/year) [51], with urinary protein excretion rates showing strong correlations with aging biomarkers including telomere shortening and aberrant DNA methylation [52]. Clinical observations highlight increased plasma activin A levels-a SASP factor-in diabetic kidneys, which promotes fibrosis and cellular senescence via SMAD2/3 pathway activation [53].

A 26-year cohort study involving 15,517 participants confirmed that the renal function decline rate in diabetic individuals is approximately double that of non-diabetic controls [51]. Regarding therapeutic interventions, umbilical cord mesenchymal stem cell (UC-MSC) therapy has demonstrated clinical potential through mechanisms involving senescence phenotype suppression and immune microenvironment modulation [54, 55]. Senolytic agents have shown efficacy in selectively eliminating senescent renal cells in preclinical studies, with early-phase clinical trials already completed [56].

Multidimensional regulatory mechanisms have been elucidated: 1) Downregulation of Sirt1 impairs mitophagy, leading to ROS accumulation and DNA damage response (DDR) activation[57]; 2) Klotho-derived peptide KP6 ameliorates renal aging by antagonizing Wnt/β-catenin signaling [58]; 3) PAQR3 over-expression suppresses PI3K/Akt pathway, reducing insulin sensitivity and promoting senescence [59]; 4) Ten-eleven translocation 2-mediated DNA hypomethylation defects cause abnormal hypomethylation at promoters of pro-fibrotic genes (TGF-β1, COL4A1), establishing epigenetic memory [60].

### 2.6 Gastrointestinal aging

Similar to other physiological organs, the human stomach and intestines undergo age-related decline in physiological function. T2DM exacerbates gastrointestinal aging, marked by higher rates of constipation, diarrhea, and gastroparesis compared to non-diabetic individuals [61]. A cross-sectional study shows 75% of T2DM patients exhibit gastrointestinal dysfunction versus 30% in controls [62]. Furthermore, T2DM patients demonstrate more pronounced gastrointestinal aging biomarkers, including gut microbiota dysbiosis (Table 1), shortened telomeres in intestinal epithelial cells, and elevated oxidative stress markers [63]. Compared to non-diabetic individuals, T2DM patients exhibit increased intestinal permeability and greater intestinal barrier impairment [64].

IR in T2DM patients impairs autophagy and increases mitochondrial oxidative stress in intestinal epithelial cells by disrupting the PI3K/Akt/mTOR pathway. The upregulation of PAQR3, a negative regulator of PI3K, inhibits insulin signaling and worsens intestinal epithelial senescence [90]. Meanwhile, chronic hyperglycemia activates nuclear factor kappa B(NF-?B) pathway, driving pro-inflammatory cytokine release and barrier dysfunction [91]. Murine models reveal hyperglycemia-induced GLUT2-dependent transcriptional reprogramming disrupts tight/adherens junctions. Genetic studies link T2DM-associated loci (TCF7L2, RFX6) to impaired intestinal stem cell function and epithelial regeneration. For instance, reduced RFX6 expression not only impairs β-cell function but also disrupts the development of intestinal endocrine cells, delaying mucosal repair [92]. Non-coding mutations (e.g., rs7903146) inhibit Wnt signaling, hindering epithelial regeneration [93].

Recent studies highlight that gut microbiota metabolites (e.g., secondary bile acids) modulate host epigenetics via FXR, while microbial lipopolysaccharide triggers TLR4-mediated systemic inflammation, perpetuating a "gut-metabolism-aging" cycle [94, 95]. These findings underscore T2DM as a potent accelerator of gastrointestinal aging through intertwined metabolic, genetic, and microbial pathways.

**Table 1:** Altered intestinal flora in diabetic patients.

Population	Comparation	Outcome	Study design
**T2DM (n = 21)**	HC (n = 30)	Proinflammatory bacteria and pathogenic bacteria increased, anti-inflammatory bacteria and probiotics decreased.	Cross sectional [65]
**T2DM cynomolgus monkeys (n = 7)**	HC (n = 30)	There were 17 species of low abundance that displayed considerable dissimilarities in both groups.	Cross sectional [66]
**PTB-DM (n = 13)**	HC (n = 13)	PTB (n = 13)	The composition of the intestinal flora is seriously altered in PTB-DM while less severely damaged in PTB patients.
**Cross sectional [67]**	T2DM (n = 48)	HC (n = 46)	Gut microbiota analysis linked Escherichia-Shigella to T2DM, contrasting with Lacticaseibacillus's inverse correlation.
**Cross sectional [68]**	T2DM (n = 1, 590)	non-T2DM	(n = 9, 010)
**DI-GM negatively links to T2DM and IR, partially mediated by BMI and inflammation.**	Cross sectional [69]	T2DM with microvascular complications, (n =22)	T2DM alone
**(n =27)**	Clostridia and Negativicutes were elevated in the study group versus controls, whereas Gammaproteobacteria, Bacilli, and Verrucomicrobia declined.	Cross sectional [70]	T2DM (n = 1,851)
**Prediabetes**	(n = 2,770)	NG (n = 2,277)	T2DM-linked microbial shifts spanned 19 taxonomically distinct species (FDR <0.10), including elevated Clostridium bolteae and diminished Butyrivibrio crossotus.
**Cross sectional [71]**	T2DM (n = 14)	HC (n = 7)	Prolonged T2DM may deplete gut probiotics, potentially triggering RLR-NF-?B inflammatory signaling and implicating microbiome-inflammation interplay in disease progression.
**Cross sectional [72]**	Prediabetes (n = 57)	HC (n = 60)	Prediabetic individuals exhibited reduced gut flora diversity versus healthy controls, featuring 9 depleted and 14 enriched bacterial genera.
**Cross sectional [73]**	T2DM (n = 18)	Prediabetes (n = 24)	HC (n = 52)
**Between pre-T2DM and healthy, and T2DM and healthy, respectively, ten and nine bacterial taxa varied in abundance**	Cross sectional [74]	T2DM (n = 183)	HC (n = 74)
**Genus-level intestine marker bacteria were Bifidobacterium, Streptococcus, and Blautia.**	Cross sectional [75]	T2DM (Han population) (n = 12)	T2DM (Dai population) (n = 12)
**HC (Han population) (n = 8)**	HC (Dai population) (n = 10)	T2DM patients showed elevated Bacteroidetes (Han group) and Proteobacteria (Dai group), with concurrent Firmicutes reduction in both populations versus healthy controls.	Cross sectional [76]
**T1DM (n = 10)**	T2DM (n = 10)	HC (n = 13)	T2DM patients exhibited reduced Verrucomicrobia abundance versus healthy controls.
**Cross sectional [77]**	T2DM (n = 461)	non-T2DM (n = 119)	The Firmicutes/Bacteroidetes ratio was higher in T2DM than non-diabetics.
**Cross sectional [78]**	T2DM (n = 74)	HC (n = 76)	T2DM exhibited distinct gut microbiota profiles marked by reduced a-diversity, an elevated Firmicutes/Bacteroidetes (F/B) ratio, and divergent -diversity.
**Cross sectional [79]**	T2DM (n = 96)	non-T2DM (n = 923	T2DM's gut microbiota featured elevated Bifidobacterium/Streptococcus and reduced Roseburia/Blautia.
**Cross sectional [80]**	T2DM (n = 46)	non-T2DM (n = 48)	Pakistani T2DM patients showed increased circulating LBP/TLR pathways and unique intestinal flora characteristics (e.g., enriched Libanicoccus/Parolsenella).
**Cross sectional [81]**	T2DM (n = 105)	HC (n = 45)	T2D patients showed depleted butyrate-producing bacteria alongside enriched pathogenic Enterobacteriaceae and Fusobacterium.
**Cross sectional [82]**	T1DM (n = 40)	T2DM (n = 40)	HC (n = 40)
**B.longum and F.prausnitzii declined in both T1DM and T2DM, whereas A.hallii rose in T1DM but fell in T2DM. Conversely, A.muciniphila decreased in T1DM and increased in T2DM.**	Cross sectional [83]	T2DM (n = 75)	non-T2DM (n = 141)
**Diabetic gut microbiota featured elevated proinflammatory Proteobacteria and reduced butyrate-producing Clostridiales.**	Cross sectional [84]	T2DM (n = 60)	Prediabetes (n = 60)
**non-T2DM (n = 60)**	T2DM showed increased Prevotella/Alloprevotella compared to controls, while Paraprevotella was reduced in both T2DM and PreDM.	Cross sectional [85]	T2DM (Denmark)
**(n = 241)**	T2DM (India) (n = 157)	NG (Denmark) (n = 138)	NG (India) (n = 137)
**T2DM patients exhibited elevated Lachnospiraceae OTUs alongside reduced Subdoligranulum and Butyricicoccus.**	Cross sectional [86]	T2DM (n = 14)	non-T2DM (n = 23)
**While major bacterial phyla/families showed no intergroup differences, 16 OTUs exhibited significant divergence.**	Cross sectional [87]	T2DM (n = 307)	Prediabetes (n = 506)
**UT2D (n =154)**	NG (n = 735)	Actinobacteria, Firmicutes, and Synergistetes inversely linked to T2D, while Lentisphaerae correlated positively	Cross sectional [88]
**T2DM (n = 7)**	non-T2DM (n = 7)	Marked disparities in Firmicutes and Clostridia abundance emerged between patients and controls.	Cross sectional [89]

HC: Healthy controls; T2DM Type 2 diabetes mellitus; T1DM: Type 1 diabetes mellitus; PTB: Pulmonary tuberculosis; DI-GM: Dietary index for gut microbiota; NG: Normoglycemic; UT2D: Undiagnosed type 2 diabetes

### 2.7 Pancreatic aging

Emerging evidence indicates accelerated pancreatic aging in individuals with T2DM, characterized by premature declines in β-cell function and early-onset glucose intolerance compared to non-diabetic populations [96]. Clinically, this manifests as progressive insulin secretory defects and elevated senescence-associated biomarkers, including heightened β-galactosidase (SA-β-gal) activity in diabetic mouse models [97]. Paradoxically, cross-sectional human studies report reduced plasma β-Gal activity in T2DM patients aged >65 years versus age-matched controls [98]. Elevated SASP factors in eripheral blood correlate with IR and β-cell dysfunction, reinforcing the link between metabolic stress and cellular aging [99].

The decline in β-cell function arises from multifactorial mechanisms, including chronic hyperglycemia, oxidative stress, and lipotoxicity. In vivo studies identify β-cell dedifferentiation with FoxO1 deficiency as a unifying pathway across metabolic stress models [100]. Genetically, GWAS reveal that T2DM-associated loci predominantly disrupt β-cell development and insulin secretion rather than peripheral insulin action [101]. A recent single-cell sequencing study has unveiled a single-cell atlas of pancreatic islets, acinar, and endothelial cells in diabetes, identifying metabolic genes such as ACE and PASK as key players in disease-related pathways [102]. Longitudinal data further link WFS1 variants to progressive insulin secretion loss, underscoring genetic susceptibility to β-cell failure [103]. Prospective intervention studies highlight the reversibility of metabolic dysfunction in T2DM. For instance, intensive dietary modifications have been shown to restore β-cell function and insulin sensitivity partially, implying bidirectional crosstalk between metabolic disturbances and pancreatic aging [104].

### 2.8 Hepatic aging

Mounting evidence highlights the relationship between T2DM and accelerated liver aging, with the liver serving as a central metabolic organ vulnerable to diabetes-driven senescence. Epidemiologically, T2DM patients exhibit distinct hepatic aging markers compared to non-diabetic individuals. Cross-sectional studies reveal significantly elevated liver fibrosis scores and steatosis severity in T2DM populations [105]. Notably, abnormal liver function markers (e.g., ALT/AST elevation) are more prevalent in T2DM patients and synergistically interact with metabolic syndrome components to accelerate hepatic aging [106]. Large-scale cohort studies from the UK Biobank and Michigan Genomics Initiative (MGI) further demonstrate that the PNPLA3 genotype combined with diabetes markedly increases the risk of fibrosis progression and hepatic decompensation in non-alcoholic fatty liver disease (NAFLD) patients [107], highlighting a gene-environment interplay in liver aging.

Mechanistically, T2DM exacerbates liver aging through glucotoxicity, lipotoxicity, and multifactorial pathways. Chronic hyperglycemia and free fatty acid overload activate hepatic stellate cells, driving fibrogenesis via TGF-β/SMAD signaling, while inflammation (NF-?B-mediated cytokine release) and apoptosis (caspase-3 activation) exacerbate tissue damage concomitantly. Sinusoidal capillarization-characterized by endothelial dysfunction and disrupted hepatic microcirculation-further impairs metabolic homeostasis [108]. Epigenetic perturbations, such as non-coding DNA mutations, disrupt metabolic gene expression (e.g., PGC-1a) by impairing transcription factor binding, thereby aggravating oxidative stress and fibrotic remodeling [109].

**Figure 3.**
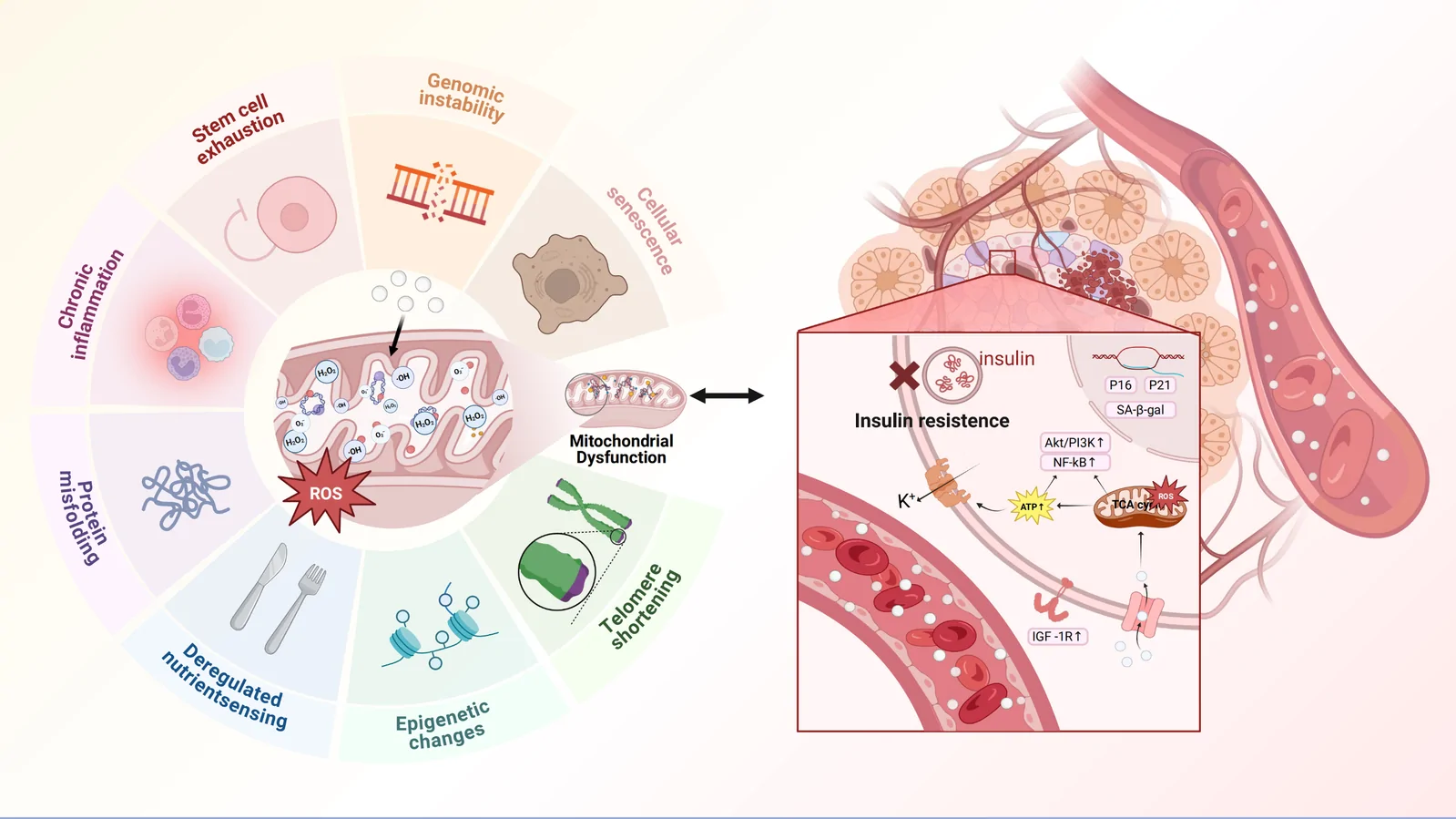
**Insulin resistance and oxidative stress as the core of the classical mechanism of promoting aging.** T2DM is typically characterized by chronic hyperglycemia and IR. Mechanistically, high glucose induces cellular production of ROS (O2-, H2O2, and -HO) leading to mitochondrial dysfunction and DNA damage (telomere shortening), and triggers persistent epigenetic changes, chronic inflammation, protein misfolding, and cellular senescence through NF-?B, p38/MAPK, and TORC1 (mTOR)-mediated pathways. Inflammation, protein misfolding and cellular senescence through NF-?B, p38/MAPK and TORC1 (mTOR)-mediated pathways. Activation of the NF-?B signaling pathway by oxidative stress inhibits the normal tyrosine phosphorylation of the insulin receptor InsR and the insulin receptor substrate IRS. It reduces the gene and protein expression of GLUT4, ultimately leading to IR and hyperinsulinemia. On the one hand, target cells chronically exposed to high insulin concentrations promote the expression of cellular senescence markers through enhanced insulin-like growth factor (IGF) transactivation, which leads to cellular senescence; on the other hand, high insulin concentrations negatively affect the cell division program through downstream PI3K/Akt and mTOR signaling, which leads to cellular senescence. NF-?B: Nuclear factor kappa B; IGF-1R:Insulin-like growth factor 1 receptor; PI3K/Akt: Phosphoinositide 3-Kinase/protein kinase B; mTORC2: Mechanistic target of rapamycin complex 2; SA-β-gal:Senescence-associated β-galactosidase.

In conclusion, diabetes accelerates multiorgan aging through the synergistic action of IR and oxidative stress, which propagate tissue homeostatic collapse and cellular senescence through interrelated molecular pathways (Fig. 3). Chronically elevated glucose and metabolites like free fatty acids disrupt insulin signaling pathways via oxidative stress-mediated inhibition of insulin receptor substrate-1 phosphorylation and PI3-kinase redistribution [110]. In obese individuals, hyperglycemia and hyperlipidemia stimulate ROS generation through NADPH oxidase (NOX) activation, impairing Akt activity and exacerbating skeletal muscle insulin resistance [111]. This self-perpetuating cycle further compromises metabolic regulation while promoting β-cell failure through sustained oxidative damage. Emerging evidence implicates Netrin-4, a laminin-related secretory protein, in mediating obesity-driven IR and redox imbalance [112]. Diabetes-related complications are also the result of accelerated aging in T2DM, and significant co-morbidity characterizes the mechanism of complications in different organs. Experimental models of T2DM have shown that the DDR pathway hyperactivation is strongly associated with fibrosis in organs such as the kidneys and lungs. This is further supported by human longitudinal studies, which have shown that cumulative DNA damage and SASP secretion precede the onset of nephropathy and restrictive lung disease in prediabetic and diabetic populations [113]. Significantly, aging biomarkers, including epigenetic aging traits, independently predict long-term cardiovascular complications in patients with T2DM [114]. Although these mechanisms overlap with natural aging processes, such as age-related metabolic dysfunction and chronic low-grade inflammation, diabetes amplifies the impact of these mechanisms through disease-specific stressors [115]. For example, intrinsic aging and environmental factors (e.g., ultraviolet exposure, trauma) exacerbate baseline IR and oxidative stress, whereas T2DM introduces additional pathological factors such as glucose toxicity and AGEs deposition. These factors converge to disrupt proteostasis and contribute to immune damage.

## 3 Diabetic-Specific Comorbid Aging Pathways

While IR and ROS form the core axis driving diabetes-associated multiorgan aging, their nonspecific roles in physiological aging necessitate a deeper exploration of T2DM-specific pathological amplifiers. Beyond these shared mechanisms, diabetes introduces unique molecular stressors-distinct from age-related metabolic drift or environmental insults-that synergize with IR-ROS cascades to accelerate tissue dysfunction. These disease-specific pathways, rooted in chronic hyperglycemia and dysregulated protein homeostasis, manifest as organ-divergent yet interconnected pathologies. The following sections delineate these T2DM-exclusive pro-aging mechanisms, including glucotoxicity, AGEs toxicity, immunoinflammatory senescence, and protein amyloidosis, which collectively define the accelerated aging trajectory in diabetes.

### 3.1 Glucotoxicity

Glucotoxicity refers to impaired cellular function by prolonged hyperglycemia, which affects pancreatic β-cells and accelerates tissue aging throughout the body [116]. In pancreatic β-cells, sustained hyperglycemia disrupts fat metabolism, which, together with lipotoxicity, leads to "glycolipotoxicity" - a key cause of β-cell death and aging [117]. We summarize the relevant studies on glycolipotoxicity promoting islet cell senescence death or dysfunction as follows (Table 2). While lipid overload in T2DM is often secondary to comorbidities like obesity, experimental evidence highlights glucose as the primary catalyst: elevated glucose amplifies sterol regulatory element-binding protein (SREBP) activity, promoting saturated fatty acid accumulation and shortening cellular lifespan in model organisms [118]. What's more, high fat alone does minor damage to β-cells without high blood glucose to go along with it, suggesting that it is glucotoxicity that is at the heart of diabetes-associated aging [119].

**Table 2:** Mechanism of glycolipotoxicity leading to apoptosis and dysfunction of β-cells.

Cellular outcome	Key events	Core node(s)	Materials	Reference
**Cell death**	Oxidative stress promotes the expression of BH3-only proteins	ROS, Bim, Puma	Mice deficient for Bim and Puma	[120]
**Cell death**	Up-regulated (TNFR) -5 binds to the corresponding ligand (CD40L) to activate the STAT1 or NF-?B pathway	(TNFR) -5, STAT1, NF-?B	INS-1 β-cells, C57Bl/6 mice	[121]
**Disfunction and apoptosis**	CBP mediates acetylation of HSF1 to inhibit its activity	HSF1	Human islets, INS1832/13 cells	[122]
**Disfunction and apoptosis**	Inhibition of miR-299-5p leads to up-regulation of Trp53 effector Perp	miR-299-5p, Perp	Mouse and rat pancreatic β-cell lines	[123]
**Disfunction and apoptosis**	Overexpression of GRP75 impairs mitochondrial membrane potential, increases mitochondrial Ca2+ levels and ROS production, enhances ER-mitochondrial contact and induces apoptosis	GRP75, Ca2+, ROS	Mouse islet and rat insulinoma cells	[124]
**Cell death**	Overexpression of chREBPβ in response to glycolipid toxicity	ChREBPβ	INS-1-derived 832/13 cells	[125]
**Cell death**	Mig6 negative feedback inhibits EGFR signal transduction	Mig6, EGFR signaling	832/13 INS-1 beta cells, wistar rats, human islets	[126]
**Autophagy**	Sphingosine kinase 1 is involved in palmitate-induced autophagy in INS-1 β-cells	SphK1, LC-3-II	INS-1 β cells (clone 368)	[127]

ROS: Reactive oxygen species; BH3: Bcl-2 homology domain 3; NF-?B: Nuclear factor kappa B; STAT: Signal transducer and activator of transcription; TNFR: Tumour necrosis factor receptor; GRP: Glucose regulated protein; ChREBPβ: Carbohydrate response-element binding protein; HSF1: Heat shock factor protein 1; CBP: cAMP response element binding protein; EGFR: epidermal growth factor receptor; Mig6: mitogen-inducible gene 6; SphK: sphingosine kinase

Glucotoxicity triggers β-cell decline through multifaceted pathways. Chronic hyperglycemia suppresses Beta2/NeuroD expression via miR-30a-5p and Pgc-1a overexpression, impairing β-cell differentiation and insulin secretion [128]. Concurrently, glucose-driven oxidative stress generates excessive ROS, destabilizing mitochondrial function and activating pro-apoptotic signals such as Bim and Puma via endoplasmic reticulum stress [120, 129] The JNK/β-catenin pathway further exacerbates apoptosis through Tetraspanin-2 upregulation, while mTOR-dependent upregulation of senescence markers (e.g., SA-β-gal, p16) confirms glucotoxicity's direct link to cellular aging [130, 131].

The dangers of glucotoxicity are not limited to the pancreas. In the cardiovascular system, hyperglycemia impairs cardiac stem cell repair and leads to premature endothelial cell senescence through activation of the ASK1 and p53 signaling pathways, as well as elevated levels of plasminogen activator inhibitor-1 (PAI-1) [131-133]. In the liver, chronic hyperglycemia overexpresses the carbohydrate response element binding protein (ChREBPβ), leading to excessive glycogen accumulation and [125, 134] IR, while the SGP130 protein secreted by hepatic stellate cells stimulated by hyperglycemia in NAFLD exacerbates hepatic inflammation and fibrosis through IL-6 trans-signaling [135]. The skeletal system is similarly affected. Hyperglycemia induced iron death and mitochondrial dysfunction in bone marrow mesenchymal stem cells (BMSCs), ultimately leading to bone microstructural destruction and attenuation of bone formation [136]. In osteoblasts, high glucose promotes elevated levels of ATF3 protein in osteoblasts, inducing iron death in osteoblasts, ultimately leading to decreased osteogenic capacity and bone cortical loss [137]. In addition, activation of the cGAS/p-STING pathway in diabetes models suggests that hyperglycemia is also associated with neuroinflammation and cognitive decline [138].

The studies mentioned indicate that glycotoxicity has widespread harmful effects on the body, rather than being solely a problem for β-cells. Therefore, we propose that the concept of glucotoxicity needs to be re-evaluated. However, there is currently a lack of direct evidence regarding how glycotoxicity influences overall aging. More research is needed to explore its connection with epigenetic changes, disruptions in protein homeostasis, and other aging-related characteristics. By reconceptualizing the role of glucotoxicity in diabetic complications, we might develop new strategies for delaying diseases associated with aging.

### 3.2 Toxicity of AGEs

AGEs originate through non-enzymatic glycation and oxidative modification of reducing sugars, lipids, proteins, and nucleic acids [139]. Under chronic hyperglycemic conditions, AGE-RAGE (Receptor for advanced glycation end products, RAGE) interactions impair mitochondrial electron transport chain functionality even in normoglycemic individuals, triggering redox-sensitive signaling cascades that amplify ROS generation and establish persistent oxidative stress. This metabolic disturbance creates a self-perpetuating cycle in which oxidative stress accelerates further glycation and oxidation of biomolecules, thereby increasing AGEs synthesis [140]. Notably, hyperglycemia-induced ROS production enhances NF-?B-mediated transcriptional upregulation of RAGE expression, establishing a pathological feedback loop [141].

As potent senescence accelerators, AGEs exert direct tissue-damaging effects through multiple mechanisms. Their capacity to form irreversible crosslinks with structural proteins drives both cutaneous aging and cardiovascular pathology by altering extracellular matrix biomechanics [142, 143]. In vitro studies demonstrate that AGE-modified collagen matrices impair tissue elasticity through multifaceted mechanisms: restricting fibrillar sliding dynamics, disrupting cell-matrix adhesion complexes, modifying mechanotransduction via stretch-activated ion channels and primary cilia, and compromising nuclear responses to mechanical stimuli [144]. Renal pathophysiology reveals distinct AGE-mediated aging pathways, where RAGE activation induces endoplasmic reticulum stress and p21-dependent premature senescence in diabetic nephropathy, while concurrent inhibition of SREBP-cleavage activating protein (SCAP) signaling promotes tubular lipid accumulation [145, 146]. Age-related renal decline correlates strongly with AGE-induced upregulation of senescence markers (p53/p21) and oxidative damage indicators (4-HNE) [147]. Emerging evidence implicates the AGE-RAGE axis in cerebral aging through blood-brain barrier disruption, proteotoxic aggregation, and potentiation of neuronal apoptosis, as comprehensively analyzed in recent neurogerontological reviews [148].

The diabetic microenvironment exacerbates stem cell aging through AGE-mediated dysregulation of cellular differentiation. While suppressing osteogenic potential in BMSCs, periodontal (PDLSCs), neural (NSCs), and tendon stem cells (TSCs), AGEs paradoxically enhance adipogenesis in adipose-derived stem cells (ADSCs) via RAGE-dependent endothelial interactions [149]. The specific mechanisms by which AGEs lead to the abnormal differentiation of various types of stem cells remain to be elucidated. However, AGE/RAGE and Wnt/β-catenin signaling have been shown to be essential pathways that inhibit the osteogenic differentiation of ASCs [150]. AGEs affect the osteogenic differentiation of ASCs through autophagy in the diabetic microenvironment [151].

### 3.3 Immunoinflammatory aging

It is well known that chronic inflammation in T2DM involves elevated inflammatory mediators and activation of NOD-like receptor pyrin domain-containing 3 (NLRP3) inflammatory vesicles. Emerging evidence highlights distinct immunometabolic mechanisms underlying diabetes-accelerated aging, diverging fundamentally from physiological inflammaging. While age-related chronic low-grade inflammation primarily arises from immune system senescence and homeostatic dysregulation, T2DM establishes a self-perpetuating "metabolic imprint" through pathological crosstalk between nutrient-sensing pathways and immune networks. This unique immunoinflammatory aging phenotype manifests through two interconnected axes.

First, metabolic abnormalities are the initiating factors of immunoinflammatory aging in T2DM. The triad of hyperglycemia, IR, and glucolipotoxicity initiates a feed-forward inflammatory cascade response through activation of NLRP3 inflammatory vesicles in pancreatic islets, particularly through macrophage-derived IL-1β [152].Obesity-driven fat tissue dysfunction releases fatty acids and cytokines (e.g., IL-6, TNF-a), reprogramming liver Kupffer cells and blood monocytes toward pro-inflammatory states [153]. Gut microbiota imbalances amplify systemic inflammation through endotoxin leakage and oxidative stress-induced damage associated molecular patterns (DAMPs), sustaining toll-like receptor 4 (TLR4) immune activation [154].

Second, an imbalance of the adaptive immune system accelerates the T2DM aging process. Elevated glucose levels disrupt Th17/Treg cell balance by overactivating IL-21/TGF-β signals, causing excessive IL-17/IFN-? production [155]. This links directly to T2DM-associated SASP - a hallmark of aging cells that release inflammatory molecules, growth factors, and tissue-remodeling enzymes. SASP drives chronic disease progression by altering tissue environments and transmitting senescence signals [156]. Unlike natural aging, T2DM-related SASP activation depends on metabolic stress (e.g., DDR pathway activation) and persists independently of age [157, 158]. Notably, hyperinsulinemia and obesity trigger fat/liver cell aging via inflammatory SASP and cell cycle blockers [159, 160]. Epigenetic changes further lock immune cells into chronic pro-inflammatory states, creating lasting "metabolic memory" [161].

However, the causal relationship between T2DM and the sequence in which the immune-inflammatory response occurs remains controversial in the current academic community. Longitudinal cohort studies have shown that elevated serum IL-1β levels predicted the risk of diabetes mellitus at 5 years (HR=1.34, 95%CI 1.12-1.61), suggesting that inflammation may precede overt metabolic disorders [162], while Mendelian randomization analyses have confirmed that shortened telomere length in lymphocytes is associated with a faster glycemic progression in T2DM [163]. This bidirectional interaction creates a unique “metabolic-immune senescence loop”: T2DM promotes the expression of senescence biomarkers and immune system dysregulation, triggers inflammatory responses, and promotes cellular senescence, which in turn leads to chronic low-grade inflammation and immune dysfunction through the secretion of SASPs, and ultimately forms a self-sustaining pro-senescence network. This pathological feature provides an important theoretical basis for the development of intervention strategies targeting diabetes-specific senescence pathways.

### 3.4 Protein amyloidosis

Protein amyloidosis, a hallmark of aging driven by protein misfolding and aggregation, contributes to neurodegenerative disorders and organ dysfunction [164]. In T2DM, the most extensively studied proteins are islet amyloid polypeptide (IAPP) and Aβ. IAPP can accumulate in pancreatic islets and may also be deposited throughout the body, including the heart, brain, kidneys, and the endothelial cells lining blood vessels. In the brain, interactions between the amyloidogenic core regions of human IAPP and Aβ increase the likelihood of these proteins forming toxic β-barrel oligomers, which may contribute to the co-progression of AD and T2DM [165].

IAPP is produced by pancreatic β-cells alongside insulin and can form toxic aggregates under diabetic conditions, including hyperglycemia, oxidative stress, and glucolipid toxicity [166]. These aggregates destabilize cell membranes and result in abnormal vesicle-like structures that are associated with vacuolization and cell death. Glycosaminoglycans on pancreatic islet cells further concentrate IAPP near the cell membrane, worsening the damage to the lipid bilayer [167]. Additionally, IAPP can directly impact pancreatic islet endothelial cells and the vascular system, leading to cytotoxicity, inflammation, capillary enlargement, and disorganization of cells surrounding the islets [168]. Beyond the pancreas, the accumulation of circulating IAPP in the brain increases levels of pro-inflammatory cytokines (such as IL-1β, IL-6, and TNF-a) and negatively affects neurological function [169]. In diabetic nephropathy, pathological deposition of IAPP occurs in the glomerular mesangium, Bowman's capsule, and vasculature, directly contributing to progressive renal dysfunction and failure [170]. Parallel studies in prediabetic rodent models reveal that circulating IAPP oligomers induce cardiac remodeling and functional impairment [171]. These oligomers disrupt calcium homeostasis, elevate reactive aldehyde levels, and amplify pro-inflammatory IL-1β production [172, 173]. Concurrent activation of the HIF1a and fructose-6-phosphate-2-kinase signaling axis further exacerbates metabolic stress and tissue damage [174].

Similarly, Aβ, a key Alzheimer's pathology, interacts bidirectionally with T2DM. This interaction amplifies cerebrovascular inflammation and Aβ deposition, impairing vascular integrity and worsening cognitive decline in patients with comorbid diabetes and Alzheimer's [175]. Notably, Aβ accumulation in T2DM arises independently of hyperglycemia but correlates mechanistically with disrupted brain insulin signaling [176]. IR reduces kinase activity in insulin receptors and insulin-like growth factor-1 receptors, enhancing Aβ42 influx into the brain while suppressing cerebral insulin levels. Downstream of this pathway, phosphorylation of serine/threonine kinase AKT (pT308AKT1/total AKT1) demonstrates a positive association with the amyloid burden [177, 178]. Intriguingly, Aβ deposits are also detected in diabetic hearts and pancreases, though their causal relationship to aging remains unresolved [179]. Collectively, T2DM and AD form a self-perpetuating cycle: metabolic dysfunction exacerbates Aβ-driven neurovascular damage, while amyloid pathology further disrupts insulin signaling. This convergence of metabolic and neurodegenerative aging mechanisms highlights the urgency of developing therapies targeting shared amyloid-mediated pathways in diabetes.

**Figure 4.**
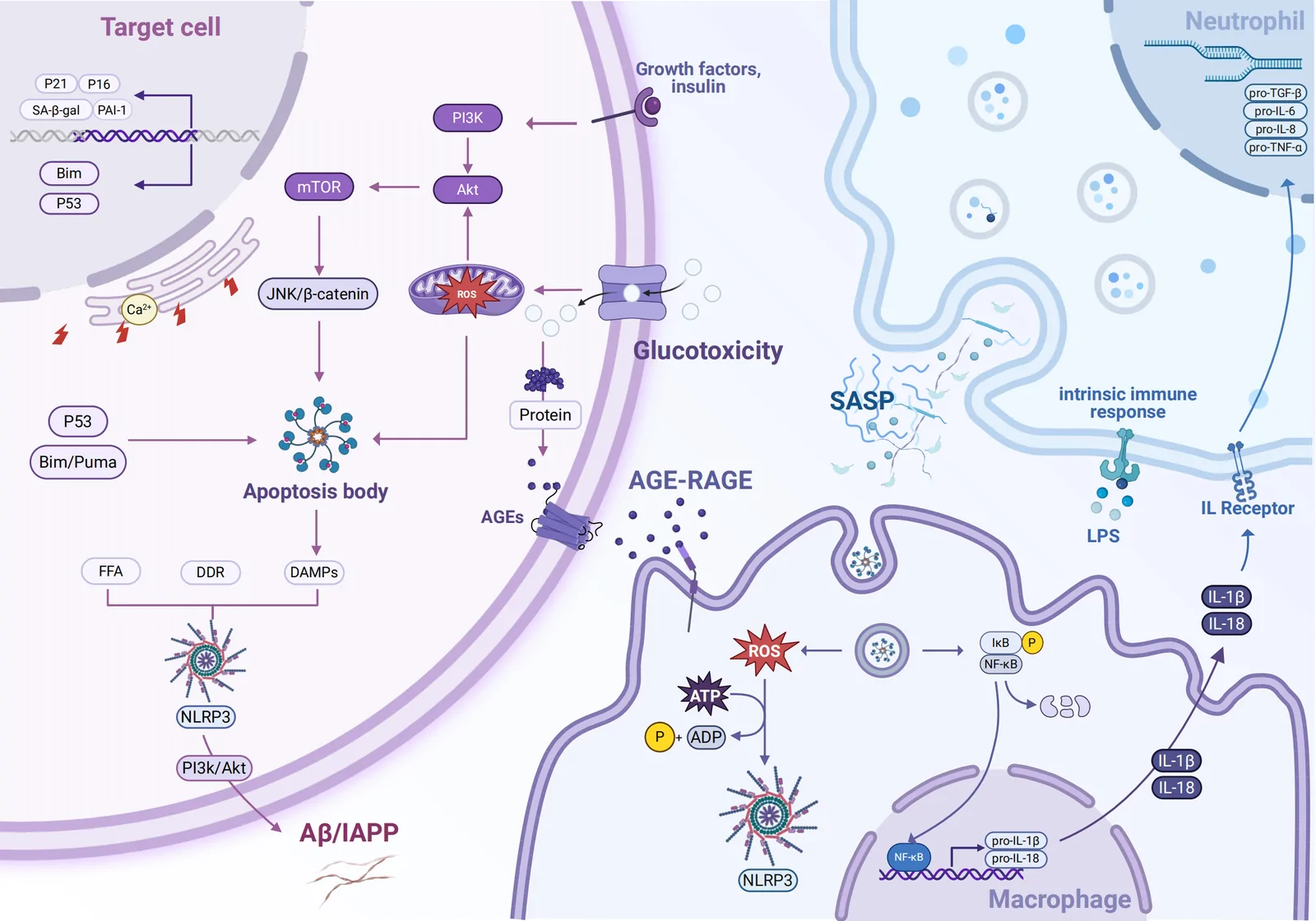
**Molecular mechanisms linking glucotoxicity, AGEs toxicity, immunoinflammatory senescence, and protein amyloidosis.** This figure illustrates the key molecular pathways and crosstalk in the diabetes-specific promotion of aging. Under conditions of glucotoxicity, AGEs bind to their receptor, activating pro-inflammatory and oxidative pathways. This triggers the p53/P21/P16 axis, leading to cell cycle arrest and SA-β-gal expression. Senescent cells exhibit the SASP, releasing inflammatory cytokines (e.g., IL-18, IL-1β) via NLRP3 inflammasome activation. Concurrently, dysregulated growth factor signaling modulates the PI3K/Akt pathway, influencing survival and metabolic responses. Accumulating pathological aggregates (e.g., Aβ/IAPP) and Bim-mediated apoptosis further exacerbate cellular damage. The interplay between AGE-RAGE signaling, intrinsic immune responses, and Wnt/β-catenin pathways underscores a network driving chronic inflammation, metabolic dysfunction, and age-related pathologies. AGEs: Advanced glycation end-products; RAGE: Receptor for advanced glycation end products; Aβ: Amyloid-beta; IAPP: Islet amyloid polypeptide; LPS: Lipopolysaccharide; DAMP: Damage-associated molecular patterns; FFA: Free fatty acids; IL: Interleukin; TGF: Transforming growth factor-β; TNF: Tumor necrosis factor; ATP: Adenosine triphosphate; SASP: Senescence-associated secretory phenotype; NLRP3: NOD-like receptor pyrin domain containing 3; PI3K/Akt: Phosphoinositide 3-Kinase/protein kinase B; JNK: Jun N-terminal kinase.

### 3.5 Interactive network of diabetes-promoted aging mechanisms

Diabetes accelerates aging through a self-amplifying "metabolism-inflammation-aging" network centered on four hubs: ROS, NLRP3, RAGE, and SASP (Fig. 4). Hyperglycemia generates AGEs that bind RAGE, activating ROS/NF-?B to amplify glucotoxicity and senescence markers (p16, p21, SA-β-gal) [131]. This cascade disrupts the blood-brain barrier, promoting Aβ deposition and T2DM-Alzheimer's comorbidity [148]. NLRP3 inflammasomes drive IAPP aggregation in pancreatic islets, while circulating IAPP spreads inflammation to kidneys/heart via macrophage recruitment and local NLRP3 activation [180]. Chronic IL-1β-driven inflammation further aggregates IAPP/Aβ, which reciprocally sustains microglial activation. ROS integrates glucotoxicity and AGE effects, NLRP3 bridges metabolism to SASP/IL-1β release, and RAGE mediates AGE-neurodegeneration crosstalk [181]. SASP propagates senescence signals systemically. These synergistic interactions culminate in β-cell failure, vascular sclerosis, hepatic fibrosis, and neurodegeneration - hallmarks of diabetes-associated premature aging.

In summary, diabetes accelerates aging through dynamic crosstalk between metabolic stress, chronic inflammation, and proteostasis failure. Therapeutic targeting of network hubs could disrupt this self-perpetuating system, offering novel strategies to delay aging and prevent multi-organ complications in diabetic patients.

## 4 Strategies for treating diabetes: From the perspective of aging regulation

Diabetes and aging intersect through shared mechanisms like cellular senescence and chronic inflammation, resulting in common phenotypes such as sarcopenia and adipose redistribution. These parallels position anti-aging strategies as viable therapeutic avenues for diabetes. While aging remains irreversible, interventions including caloric restriction, exercise, and pharmacological agents demonstrate efficacy in slowing its progression [182-185]. Many glucose-lowering medications enhance healthy lifespans in both diabetic and non-diabetic populations through mechanisms beyond glycemic control (Table 3). Interestingly, a growing body of research suggests that certain glucose-lowering drugs can show the same tendency to slow aging in groups that are not diabetic [186, 187]. This effect does not seem directly related to their ability to lower blood sugar levels. Instead, it is likely connected to various mechanisms, including regulating energy metabolism, inhibiting inflammatory responses, or activating cellular autophagy pathways [188]. However, this observation raises an important question: Do these drugs activate specific cellular protection and repair pathways directly, or do they improve metabolic balance indirectly, thereby benefiting crucial aspects of the aging process?

**Table 3:** Summary of clinical trials of metformin to slow the progression of aging or age-related diseases.

Populations	Interventions	Comparison	Outcome/Conclusion	Duration	Number
**390 patients receiving insulin therapy (age:30-80 years)**	Metformin hydrochloride, 850mg (n = 196)	Placebo (1-3 times daily) (n = 194)	Metformin can improve blood sugar control, reduce insulin needs, lose weight, and reduce the risk of developing large vascular disease.	4.3 years	NCT00375388 [189]
**380 patients underwent primary PCI**	Metformin hydrochloride, 500 mg (n = 191)	Placebo (n = 189)	Metformin therapy did not improve left ventricular function in nondiabetic patients undergoing primary PCI.	4 months	NCT01217307 [190]
**365 participants (age: 35-75 years; Diagnosed coronary heart disease; Prescribed statins)**	Metformin, 850 mg twice daily (n = 86)	Matching placebo (n = 87)	Insulin, HbA1c, HOMA-IR and tissue plasminogen activator were lower in metformin group. Metformin had no effect on cIMT.	18 months	NCT00723307 [191]
**38 prediabetic patients**	Metformin 500 mg tris in die (n = 19)	Placebo (n = 19)	Metformin notably enhanced metabolic parameters and insulin sensitivity in participants.	2 months	NCT01765946 [192]
**33 nondiabetic IR congestive heart failure patients (mean age, 63±7.0 years; men)**	Metformin, 2000mg/d (n=20)	Matching placebo (n=13)	Metformin significantly reduced FIRI, lowered BMI, and improved insulin sensitivity	4 months	NCT00473876 [193]
**20 patients with mild cognitive impairment or mild dementia without diabetes (age:55-80 years)**	Metformin, 2000 mg/d (n =10)	Placebo (n=10)	Metformin improves executive function and improves learning, memory, and attention.	16 weeks	NCT01965756 [194]
**14 ~70-year-old participants**	Metformin (first 6 weeks)	Placebo (second 6 weeks)	Metformin has metabolic and non-metabolic effects associated with aging. The metformin group reduced insulin secretion, insulin AUC, and 2-hour glucose.	12 weeks	NCT02432287 [195]
**100 patients with NDVCI and abnormal glucose metabolism**	Metformin and donepezil (n = 50)	Acarbose and donepezil (n = 50)	Metformin can improve cognitive function in patients with abnormal glucose metabolism and NDVCI.	*	ChiCTR-IPR-17011855 [196]
**healthy women and men (age:=65 years)**	Metformin, 1,700 mg/d (n = 46)	Placebo (n = 48)	Metformin negatively affects the muscle hypertrophy response of resistance training in healthy older adults.	14 weeks	NCT02308228 [197]
**16 older adults (=60 years)**	Metformin, 500 mg (n =8)	Placebo (n = 8)	Short-term metformin treatment only affected SC dynamics and mitochondrial free radicals, and did not affect aging and muscle mitochondrial respiration rate.	2 weeks	NCT03107884 [198]
**148 adults free of disease (age: 40-75 years)**	Metformin, 1 500 mg/d (n =148)	/	Ongoing	12 weeks	NCT04264897 [199]
**20 healthy older adults (BMI: <30, age: =60 years)**	Metformin (n = 10)	Placebo (n = 10)	Metformin can slow type I muscle fiber atrophy, reduce the proinflammatory transcriptional profile, and reduce the muscle FAP myofibroblast population and SASP markers.	26 days	NCT03107884 [200]
**9,901 participants with T2DM (aged =50 years)**	Dulaglutide (n=4,949)	Placebo (n=4,952)	After adjusting for baseline scores, the hazard of cognitive impairment was reduced by 14% with dulaglutide (HR 0.86, 95% CI 0.79-0.95; p=0.0018).	5.4 years	NCT01394952 [201]
**60,080 participants**	GLP-1 receptor agonists	Placebo	GLP-1 receptor agonists reduced the main adverse cardiovascular events by 14%, all-cause mortality by 12%, heart failure admissions by 11%, and composite kidney outcomes by 21%.	/	CRD42021259711 [202]
**61,412 participants regardless of using dietary or exercise therapy**	GLP-1 by injectable and oral agents.	Placebo	GLP-1 receptor agonist did not lower heart failure readmissions in patients with a history of heart failure and high N-terminal pro-B-type natriuretic peptide levels.	2 days - 5.4 years	CRD42021226231 [203]
**1,840 participants**	Semaglutide (n =920)	Placebo (n = 920)	Ongoing	156weeks	evoke (NCT04777396) & evoke+ (NCT04777409)[204]
**1,222 patients aged 18 to 85 years with type 2 diabetes**	Sotagliflozin	Placebo	Sotagliflozin improved symptoms within 4 months of worsening heart failure.	/	NCT03521934 [205]
**119 frail elders with diabetes and chronic kidney disease**	Empagliflozin (n = 59)	No-empagliflozin (n = 60)	We confirmed the significant effect of empagliflozin treatment on the amelioration of cognitive impairment.	6 months	NCT04962841 [206]
**7,020 patients with T2DM**	Empagliflozin (n = 4,687)	Placebo (n = 2,333)	In patients with T2DM at high cardiovascular risk, empagliflozin slowed kidney disease progression, reduced renal events, and lowered the rate of major cardiovascular outcomes and death when added to standard care.	3.1 years	NCT01131676 [207]

PCI: percutaneous coronary intervention; HOMA-IR: homeostatic model assessment of insulin resistance; cIMT: carotid intima-media thickness; IR: insulin resistance; FIRI: fasting insulin resistance index; BMI: body mass index; AUC: area under the plasma concentration-time curve; NDVCI: normalized difference vegetation change index; SC: Subcutaneous Injections; FAP: fibroblastic adipogenic progenitor; SASP: senescence-associated secretory phenotype; T2DM: Type 2 diabetes mellitus; HR: hazard ratio; CI: confidence interval; GLP-1: glucagon-like peptide-1.

Insulin sensitization and inflammatory resolution are critical interfaces between senescence regulation and diabetes progression. We believe that targeting oxidative stress and its downstream pathways is a key factor in treating diabetic aging. The first step is to target ROS generation. Mechanistically, Per-Arnt-Sim kinase(PASK) inhibition preserves FoxO3a activity to reduce ROS/RNS accumulation [208], while AGEs inhibitors (e.g., pyridoxamine) improve vascular integrity without perturbing β-cell function [209, 210]. SERPINE1 emerges as a pivotal regulator, where loss-of-function mutations delay telomere attrition and diabetes onset, contrasting with ER stress-induced SERPINE1 overexpression that accelerates senescence [211]. Sirtuin family proteins further bridge metabolic and aging pathways: SIRT1/SIRT3/SIRT6 enhance mitochondrial resilience, whereas SIRT2 deacetylates NLRP3 to resolve inflammasome-driven IR [212]. Intriguingly, low-dose nicotine activates the NAMPT-SIRT1 axis, boosting NAD+ synthesis to stabilize telomeres and mitigate oxidative stress without glycemic perturbation [213]. These are all effective therapeutic targets that can be applied to patients with T2DM. In the future, we anticipate more relevant drug development trials and clinical studies aimed at transforming diabetes management from a focus on “glycemic control” to an emphasis on “aging modification.”

Emerging anti-aging strategies that show additional benefits in improving metabolic disorders in diabetes include stem cell therapies, senolytics, and NAD+ precursor supplements [214]. The primary reason for this connection is that diabetes and the aging process share common features, including tissue repair capacity, cellular homeostasis, and a chronic inflammatory state. When the body accumulates senescent cells or experiences immune imbalance, insulin sensitivity often declines, making glycemic control more difficult [215]. In recent years, studies on senolytics have pointed to the fact that senescent cells release large amounts of pro-inflammatory factors that exacerbate IR and β-cell hypoplasia and that targeted removal of these cells can help reduce inflammation and improve the overall metabolic environment [216]. Similarly, NAD+ precursor supplementation has shown potential to enhance mitochondrial function and maintain the DNA repair system. It may also relieve mitochondrial damage and energetic disturbances common to the diabetic disease process [217].

Interestingly, many anti-aging approaches not initially designed for diabetes have shown positive effects on blood glucose levels, IR, and inflammatory markers in clinical or experimental studies. This suggests a close intersection between aging and diabetes: on the one hand, aging amplifies diabetes-related pathological processes; on the other hand, interventions targeting key pathological aspects of aging may also provide novel ideas for diabetes prevention and treatment.

## 5. Conclusion and Outlook

This review delineates the intrinsic interplay between diabetes and accelerated aging, highlighting distinct molecular pathways that diverge from natural aging processes. While sharing common mechanisms like oxidative stress and IR, diabetes-specific drivers-including glucotoxicity, the AGEs-RAGE axis, immune-metabolic-inflammatory cross talk, and protein amyloidosis-create unique aging trajectories with cross-organ convergence, particularly in cardiovascular, renal, and neurological systems. Notably, diabetic complications often manifest as organ-specific aging phenotypes. To dissect these mechanisms, future research should employ spatiotemporal multi-omics approaches: single-cell transcriptomics coupled with metabolomics could map IR-oxidative stress networks across tissues. At the same time, integrated epigenomic-metabolomic analyses may reveal how metabolic dysregulation triggers epigenetic reprogramming in aging cells.

Therapeutically, beyond conventional glucose-lowering agents, senolytics targeting inflammatory pathways and senescent cell clearance show promise, though long-term clinical validation remains critical. Emerging strategies include CRISPR-based correction of aging-related genes (e.g., SERPINE1, SIRT1) and metabolic reprogramming drugs to reverse aging signatures. Clinically, early intensive interventions using organ-protective agents like SGLT2 inhibitors should be prioritized, complemented by personalized approaches guided by aging biomarkers (telomere length, DNA methylation clocks). These advancements bridge mechanistic insights with translational applications, paving the way for dual-targeted therapies that concurrently address metabolic dysfunction and multi-organ aging in diabetes.

## Data Availability

Not applicable
